# Reevaluating basic life support termination criteria using post-arrival data in Japanese out-of-hospital cardiac arrest patients

**DOI:** 10.1016/j.ccrj.2026.100186

**Published:** 2026-05-12

**Authors:** Akira Suekane, Wataru Takayama, Koji Morishita

**Affiliations:** a Department of Acute Critical Care and Disaster Medicine, Graduate School of Institute of Science Tokyo, Institute of Science Tokyo, 1-5-45, Yushima, Bunkyo-ku, Tokyo, Japan; b Trauma and Acute Critical Care Center, Institute of Science Tokyo Hospital, Institute of Science Tokyo, 1-5-45, Yushima, Bunkyo-ku, Tokyo, Japan

**Keywords:** Basic life support termination of resuscitation rule, Out-of-hospital cardiac arrest, Serum potassium level

## Abstract

**Objective:**

Termination of resuscitation (TOR) rules for out-of-hospital cardiac arrest (OHCA) are applied worldwide. However, in some Asian countries, TOR is restricted by law or practice. We assessed the benefits of including post-arrival variables on the accuracy of the basic life support (BLS)-TOR rule in eligible patients.

**Design:**

A multicenter retrospective cohort study.

**Setting:**

A nationwide Japanese OHCA registry.

**Participants:**

We evaluated 81,234 patients recorded in a nationwide Japanese OHCA registry between 2014 and 2021 who were transported to 161 emergency hospitals. After applying exclusion criteria, 59,114 patients were included in the analysis. Among these, 43,737 patients fulfilled the BLS-TOR criteria.

**Main outcome measures:**

The primary outcome was 30-day survival and the secondary outcome was a favourable neurological status, defined as a cerebral performance category score of 1 or 2 at 30 days.

**Results:**

The 30-day survival and favourable neurological outcome rates were 0.9% and 0.1%, respectively. Multivariate analysis identified serum potassium as the strongest predictor of poor outcomes (adjusted odds ratio for survival: 0.45; 95% confidence interval: 0.42–0.49). When serum potassium thresholds were added to the BLS-TOR criteria, survival rates declined progressively—0.72, 0.40, and 0.21% at ≥5.0, ≥6.0, and ≥7.0 mEq/L, respectively. At ≥7.0 mEq/L, specificity for predicting 30-day mortality reached 0.99, and the positive predictive value was 1.00.

**Conclusions:**

Incorporating post-arrival in-hospital variables provides a novel approach to guide TOR. Serum potassium, a readily measured arrival marker, is one promising candidate.

## 1 Introduction

Termination of resuscitation (TOR) rules for out-of-hospital cardiac arrest (OHCA), proposed by the American Heart Association and the European Resuscitation Council, have been adopted in several countries.[1], [2], [3], [4] Although TOR rules aim to optimise resource allocation while preserving patient dignity,[5], [6], [7] their clinical application requires careful consideration.^8^

The basic life support (BLS) TOR rule—defined by (1) cardiac arrest (CA) not witnessed by emergency medical service (EMS) personnel, (2) no defibrillation delivered by either a public-access automated external defibrillator or by EMS personnel, and (3) no return of spontaneous circulation (ROSC) prior to hospital arrival—exhibits low specificity for predicting death in non-Western countries, where legal restrictions often prevent TOR at the scene, unlike in North America and Europe.^9^^,^^10^ In some Asian countries, including Japan, EMS personnel are not legally permitted to declare death at the scene and TOR cannot be implemented for OHCA cases except in the presence of definitive signs of death, such as brain extrusion or rigor mortis.[10], [11], [12], [13], [14] Consequently, nearly all patients with OHCA, including those with no likelihood of survival, are routinely transported to hospitals, imposing a substantial burden on medical personnel, resources, and time. Therefore, a simplified TOR rule is required for settings where most patients with OHCA are transported to the emergency department (ED). Although several new TOR rules have been proposed for the Japanese EMS system,^10^^,^[15], [16], [17] they primarily rely on pre-hospital information obtained by EMS personnel and may not accurately reflect a patient’s clinical status upon arrival at the ED. Therefore, a readily available in-hospital variable with prognostic value should be incorporated into the new TOR rules. Although blood test values available at emergency hospitals have been linked to OHCA prognosis,[18], [19], [20] they are not incorporated into the current TOR rules.

This study aimed to evaluate the effectiveness of the BLS-TOR rule for OHCA patients in Japan. Subsequently, we assessed the prognostic value of incorporating different post-arrival variables into the conventional BLS-TOR rule for these patients.

## 2 Methods

### 2.1 Study design and setting

This analysis was conducted using the Japanese Association for Acute Medicine–OHCA (JAAM–OHCA) registry, a multicenter prospective registry of OHCA in Japan.^21^ We included 81,234 patients with OHCA who were transported to 161 emergency hospitals between June 2014 and December 2021. The JAAM–OHCA registry dataset has been available since 2014; however, access to specific years is restricted depending on the timing of study proposal approval. Accordingly, the present study included patients from 2014 to 2021, which were available for analysis in this project. The study was conducted in accordance with the principles of the Declaration of Helsinki. The Institutional Review Board at the Institute of Science Tokyo (M2000-2099-4) approved the retrospective analysis of anonymised data and the requirement for written informed consent was waived.

### 2.2 Study population

We included all adult patients (>18 years) with OHCA registered in the JAAM–OHCA registry between June 2014 and December 2021. We excluded patients when (a) a physician was on board the ambulance, (b) patients received pre-hospital care from a physician, because advanced life support (ALS) interventions—including advanced cardiac life support—would already have been initiated and the BLS-TOR rule would therefore not be applicable. Additionally, we excluded patients when (c) CA was due to accidental hypothermia because the BLS-TOR rule is applicable only when no physician is involved and the standard TOR guidelines are not applicable when CA is caused by accidental hypothermia.^16^

### 2.3 Data collection

We extracted the following clinical data from the JAAM–OHCA registry: patient demographics (sex and age); event-related variables (witnessed arrest, bystander cardiopulmonary resuscitation [CPR], initial cardiac rhythm, CA location, and aetiology); ROSC upon hospital arrival; initial blood gas and laboratory parameters, including pH, partial pressure of alveolar carbon dioxide (PaCO_2_), partial pressure of arterial oxygen (PaO_2_), bicarbonate (HCO_3_), base excess, lactate levels, glucose (Glu) levels, sodium levels, and potassium levels; time course data (time of 911 call, on-site arrival, hospital arrival, first blood sampling, cessation of resuscitation, and TOR decision); and outcomes, including survival at hospital discharge and cerebral performance category (CPC) score.

The JAAM–OHCA registry is maintained by the Japanese Association for Acute Medicine in collaboration with participating emergency medical institutions nationwide. Data are prospectively entered by trained personnel at each participating site using a standardised data collection form.

### 2.4 Outcomes and definitions

The primary outcome was survival at 30 days. The secondary outcome was a favourable neurological status at 30 days and ROSC after ED admission. Based on the CPC scores, neurological status was classified as follows: Category 1, good cerebral performance; Category 2, moderate cerebral disability; Category 3, severe cerebral disability; Category 4, coma or vegetative state; and Category 5, death/brain death.^22^ A favourable neurological status was defined as a CPC score of 1 or 2. The international BLS-TOR rule specifies the following three criteria: (1) CA not witnessed by an EMS personnel, (2) no defibrillation delivered by either a public-access automated external defibrillator or by an EMS personnel, and (3) no ROSC prior to hospital arrival. Meeting all three criteria supports the termination of resuscitative efforts in patients as the likelihood of meaningful survival is extremely low.[1], [2], [3], [4] Time to withdrawal was defined as the time to cessation of resuscitation efforts. The duration of EMS-performed CPR was defined as the time from initiation of resuscitation to hospital arrival.

### 2.5 Statistical analysis

Based on whether they met the international BLS-TOR criteria on ED admission, patients were categorised into (a) fulfilled BLS-TOR and (b) non-fulfilled BLS-TOR groups. We used the analysis of variance to assess the baseline characteristics and outcomes of the two groups. For the univariate analysis, continuous and categorical variables were expressed as medians (with interquartile range [IQR], first to third quartiles) and percentages, respectively. Multivariate analysis was performed using post-arrival laboratory values, with 30-day survival and favourable neurological outcome (CPC 1 or 2) as dependent variables. ORs were calculated for each dependent variable. Variables that were clinically relevant and emerged as significant in the univariate analysis were used in the multivariate analysis (Supplementary Table 1). For estimated size and variability, we reported the sensitivity, specificity, positive predictive value (PPV), negative predictive value, and area under the receiver operating characteristic curve (AUROC), each with a 95% confidence interval (CI), for cases in which the conventional TOR rule alone was met and for those in which the TOR rule combined with a serum potassium threshold was fulfilled. R software (version 4.3.2, R Foundation for Statistical Computing, Vienna, Austria) was used for the statistical analysis. Statistical significance was set at p < 0.05.

## 3 Results

### 3.1 Details of overall cohort

**Fig. 1:**
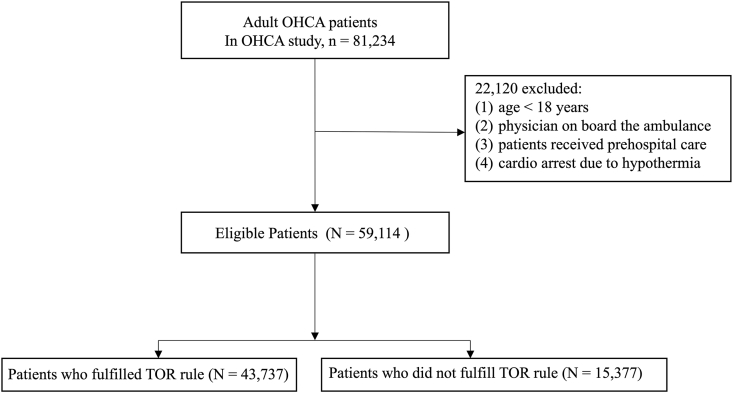
Flow chart of patient selection.

Fig. 1 shows the patient selection flowchart. Of the 81,234 patients recorded in the registry, 59,114 met the eligibility criteria and were included in the analysis. Among these, 43,737 patients (74.0%) fulfilled the BLS-TOR rule. Table 1 summarises the baseline characteristics of the patients. The median age of the participants was 77.0 [IQR, 64.0–85.0] years, and 23,960 patients (40.5%) were women. Among these eligible 59,114 patients, 25,405 CAs (43.0%) were witnessed by bystanders, and bystander CPR was performed in 25,740 patients (43.5%), and 4483 patients (7.6%) had an initial shockable rhythm. Almost half of the CA cases were of cardiovascular origin. The median duration of CPR performed by EMS before hospital arrival was 22 min [17.0–29.0]. Pre-hospital ROSC occurred in 6091 patients (10.3%). Of the 43,737 patients (74.0%) who met the BLS-TOR criteria, only 44 patients (0.1%) had an initial shockable rhythm. The distribution of CA aetiologies was comparable between groups. Patients who fulfilled the BLS-TOR criteria differed significantly from those who did not across several baseline characteristics. The BLS-TOR–fulfilled group was older (median age, 78 [IQR 66–86] vs. 73 [60–83] years) and included a higher proportion of women (43.1% vs. 33.2%). A witnessed arrest was substantially less frequent in the BLS-TOR–fulfilled group (31.3% vs. 76.0%) as was an initial shockable rhythm (0.1% vs. 28.9%). By definition, no patient in the BLS-TOR–fulfilled group achieved pre-hospital ROSC (0% vs. 39.6%). In addition, asystole was markedly more common (78.0% vs. 23.1%), whereas cardiovascular aetiology was less frequent (51.1% vs. 61.4%) in patients who fulfilled the BLS-TOR criteria.

**Table 1:** Patient characteristics.

Variables			Overall	Not fulfilled the BLS-TOR rule	Fulfilled the BLS TOR rule	p
			n = 59,114	n = 15,377	n = 43,737	
**Sex, female (%)**			23,960 (40.5)	5112 (33.2)	18,848 (43.1)	**<0.01**
**Age (median [IQR])**			77.0 [64.0, 85.0]	73.0 [60.0, 83.0]	78.0 [66.0, 86.0]	**<0.01**
**With a witness of CA (%)**			25,405 (43.0)	11,694 (76.0)	13,711 (31.3)	**<0.01**
**With bystander CPR (%)**			25,740 (43.5)	5608 (36.5)	20,132 (46.0)	**<0.01**
**CPR duration by EMS (median [IQR])**			22.0 [17.0, 29.0]	21.0 [14.0, 28.0]	23.0 [18.0, 30.0]	**<0.01**
**Initial rhythm (%)**						**<0.01**
**Vf**			4394 (7.4)	4362 (28.4)	32 (0.1)	
**pVT**			89 (0.2)	77 (0.5)	12 (0.0)	
**PEA**			14,457 (24.5)	5138 (33.4)	9319 (21.3)	
**Asystole**			37,676 (63.7)	3551 (23.1)	34,125 (78.0)	
**Pre-hospital ROSC (%)**			6091 (10.3)	6091 (39.6)	0 (0.0)	**<0.01**
**Causes (%)**	**Non-Cardio-vascular**	**Cerebrovascular**	1993 (3.4)	641 (4.2)	1352 (3.1)	
		**Pulmonary**	3355 (5.7)	878 (5.7)	2477 (5.7)	
		**Neoplasm**	1419 (2.4)	325 (2.1)	1094 (2.5)	
		**External causes**	12,408 (21.0)	2192 (14.3)	10,216 (23.4)	
	**Cardiovascular**		31,783 (53.8)	9441 (61.4)	22,342 (51.1)	**<0.01**
**ECPR (%)**			2058 (3.5)	1760 (11.4)	298 (0.7)	**<0.01**

Data are presented as unweighted numbers (percentages) of patients, unless otherwise indicated.

Abbreviations: BLS-TOR, basic life support-termination of resuscitation; IQR, interquartile range; CPR, cardiopulmonary resuscitation; CA, cardiopulmonary arrest; ROSC, return of spontaneous circulation; Vf, ventricular fibrillation; pVT, pulseless ventricular tachycardia; PEA, pulseless electrical activity; EMS, emergency medical service; ECPR, extracorporeal cardiopulmonary resuscitation.

External causes include trauma, drowning, hanging, intoxication, and other non-medical aetiologies.

### 3.2 Outcome of patients based on the BLS-TOR rule

Table 2 compares the outcomes in patients who did and did not fulfill the BLS-TOR rule. The former group had a significantly lower 30-day survival (0.9% vs. 20.5%; p < 0.01), worse neurological outcome at 30 days (0.1% vs. 11.6%; p < 0.01), and a lower rate of ROSC after hospital admission (18.7% vs. 31.4%; p < 0.01) compared to the non-fulfilled BLS-TOR group.

**Table 2:** Comparison of patients who did and did not fulfill the BLS-TOR rule.

Outcome and strata	Overall n = 59,114	Not fulfilled the BLS-TOR rule n = 15,377	Fulfilled the BLS-TOR rule n = 43,737	p	SMD
Primary outcome					
**Survival at 30 days (%)**	3567 (6.0)	3159 (20.5)	408 (0.9)	**<0.01**	0.67
**Secondary outcome**					
**Favourable CPC at 30 days (%)**	1833 (3.1)	1780 (11.6)	53 (0.1)	**<0.01**	0.50
**ROSC after arrival (%)**	13,029 (22.0)	4831 (31.4)	8198 (18.7)	**<0.01**	1.08

Data are presented as unweighted numbers (percentages) of patients, unless otherwise indicated.

Abbreviations: BLS-TOR, basic life support-termination of resuscitation; IQR, interquartile range; ROSC, return of spontaneous circulation; CPC, Cerebral Performance Category.

### 3.3 Associations with survival and favourable neurological outcome

Univariable associations between post-arrival variables and 30-day survival, stratified by BLS-TOR fulfilment, are summarised in Supplementary Table 1.

Table 3 presents the results of the multivariate analysis of all patients. Adjusted ORs for pH, PaCO_2_, PaO_2_, HCO_3_, SaO_2_, base excess , lactate, Glu, sodium, and potassium were evaluated using multivariable logistic regression analysis for 30-day survival and a favourable neurological outcome. Among these variables, serum potassium level emerged as an independent risk factor, showing the lowest adjusted odds ratio (OR) for 30-day survival [adjusted OR (95% CI): 0.45 (0.42–0.49); p < 0.01] and a favourable neurological outcome [adjusted OR (95% CI): 0.41 (0.36–0.46); p < 0.01]. Although lactate, PaO_2_, and PaCO_2_ were identified as independent risk factors, potassium showed the strongest association with poor outcomes among all variables assessed. In multivariable analyses restricted to patients who fulfilled and who did not fulfill the BLS-TOR rule, serum potassium remained independently associated with 30-day survival and a favourable neurological outcome (Supplementary Tables 2 and 3).

**Table 3:** Multivariate logistic regression analyses of post-arrival factors in all patients.

	Survival at 30 days				Favourable CPC at 30 days			
Variables	Odds ratio	Lower 95% CI	Upper 95% CI	p	Odds ratio	Lower 95% CI	Upper 95% CI	p
**pH**	1.44	0.51	3.81	0.48	1.32	0.29	4.92	0.70
**PaCO_2_**	0.99	0.99	1.00	**<0.01**	0.96	0.96	0.97	**<0.01**
**PaO_2_**	1.00	1.00	1.00	**<0.01**	1.00	1.00	1.00	**<0.01**
**HCO_3_**	0.99	0.97	1.02	0.62	1.00	0.95	1.04	0.95
**BE**	1.01	0.99	1.03	0.21	1.00	0.97	1.03	0.86
**Lactate**	1.00	0.99	1.00	**<0.01**	0.99	0.99	0.99	**<0.01**
**Glu**	1.00	1.00	1.00	0.16	1.00	1.00	1.00	0.01
**Sodium**	0.99	0.98	1.00	0.13	1.00	0.98	1.03	0.77
**Potassium**	0.45	0.42	0.49	**<0.01**	0.41	0.36	0.46	**<0.01**

Abbreviations: BE, base excess; CI, confidence interval; Glu, glucose; CPC, cerebral performance.

### 3.4 Utility of potassium in prognostication

Table 4 shows the optimal threshold, sensitivity, specificity, PPV, and negative predictive value of potassium for mortality at 30 days in patients who fulfilled the BLS-TOR rule. The 30-day survival rate in these patients was 0.93%. When potassium thresholds were applied, 30-day survival rates decreased with increasing potassium levels: 0.72% at 5.0 mEq/L, 0.40% at 6.0 mEq/L, and 0.21% at 7.0 mEq/L. Furthermore, no patient with a potassium level ≥8.5 mEq/L survived with a favourable neurological outcome at 30 days. Among patients who fulfilled the TOR rule, the addition of potassium thresholds improved the specificity and PPV of predicting the 30-day mortality. When a potassium threshold of 5.0 mEq/L was applied, specificity increased to 0.94 (95% CI: 0.93–0.95), whereas PPV remained at 0.99 (95% CI: 0.99–0.99). However, when a potassium threshold of 7.0 mEq/L was applied, specificity further increased to 0.99 (95% CI: 0.98–0.99) and PPV reached 1.00 (95% CI: 1.00–1.00). In contrast, the sensitivity decreased from 0.69 (95% CI: 0.68–0.69) at 5.0 mEq/L to 0.44 (95% CI: 0.43–0.44) at 7.0 mEq/L.

**Table 4:** Performance of potassium value for mortality at 30 days in patients who fulfilled the BLS-TOR rule.

TOR rules	The BLS-TOR rule	The BLS-TOR rule and potassium		
Criteria	Not witnessed by EMT No pre-hospital shock No pre-hospital ROSC	Not witnessed by EMT No pre-hospital shock No pre-hospital ROSC Potassium level		
**Threshold of potassium, mEq/L**		Potassium >5.0	Potassium >6.0	Potassium >7.0
**N**	43,737	20,219	16,819	12,800
**Survival at 30 days (%)**	408 (0.93)	145 (0.72)	67 (0.40)	27 (0.21)
**Sensitivity (95%CI), %**	0.78 (0.78–0.78) 0.67	0.69 (0.68–0.69) 0.67	0.57 (0.57–0.58)	0.44 (0.43–0.44)
**Specificity (95%CI), %**	0.89 (0.87–0.90) 0.75	0.94 (0.93–0.95) 0.75	0.97 (0.97–0.98)	0.99 (0.98–0.99)
**PPV (95%CI), %**	0.99 (0.99–0.99) 0.99	0.99 (0.99–0.99) 0.99	1.00 (1.00–1.00)	1.00 (1.00–1.00)
**NPV (95%CI), %**	0.21 (0.20–0.21) 0.0020	0.21 (0.20–0.22) 0.0020	0.17 (0.16–0.17)	0.13 (0.13–0.14)
**Area under the ROC curve (95%CI)**	0.83 (0.83–0.84)	0.82 (0.81–0.82)	0.77 (0.77–0.79)	0.71 (0.71–0.72)

Abbreviations: BLS, basic life support; CI, confidence interval; EMT, emergency medical team; NPV, negative predictive value; PPV, positive predictive value; ROC, receiver operating characteristic; ROSC, return of spontaneous circulation; TOR, termination of resuscitation.

**Fig. 2:**
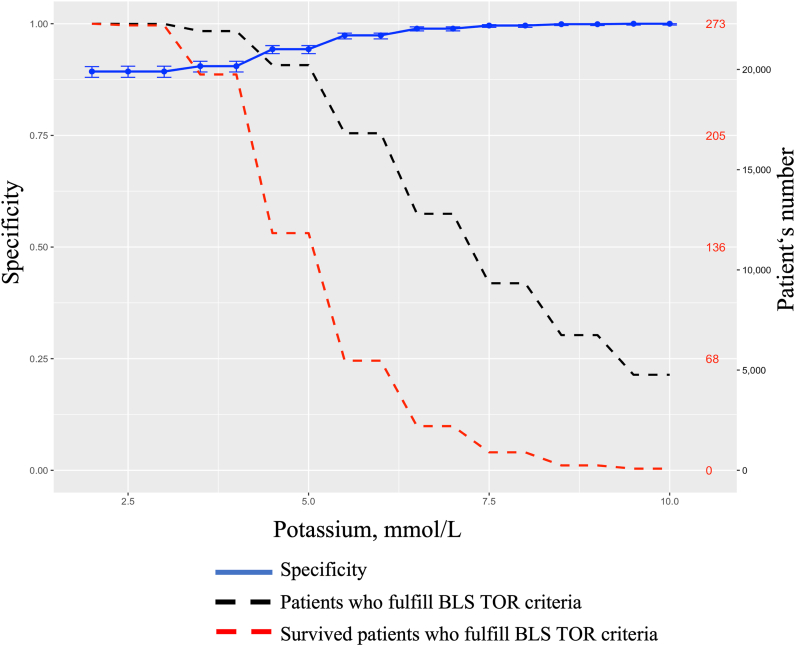
Specificity for mortality and patient distribution according to serum potassium levels. The solid blue line represents the specificity of mortality when incremental serum potassium thresholds were added to the BLS-TOR criteria. The black dashed line shows the number of patients who fulfilled the BLS-TOR criteria and their respective potassium thresholds. The dashed red line indicates the number of survivors (%). The x-axis represents the serum potassium levels (mmol/L). BLS, basic life support; TOR, termination of resuscitation. (For interpretation of the references to colour in this figure legend, the reader is referred to the Web version of this article.)

The AUROC was 0.83 (95% CI: 0.83–0.84) for the BLS-TOR rule alone and progressively decreased with higher potassium thresholds, reaching 0.71 (95% CI: 0.71–0.72) at a threshold of 7.0 mEq/L. A similar improvement in the predictive accuracy for an unfavourable CPC at 30 days was observed, as shown in Supplementary Table 4 (Supplementary Digital Content). Fig. 2 shows the relationship between the potassium levels and survival outcomes. As the potassium threshold increased, the specificity also increased, and the number of 30-day survivors decreased. Further details of the receiver operating characteristic analysis are shown in Supplementary Fig. 1.

## 4 Discussion

### 4.1 Summary of major findings

In this multicenter retrospective study, we analysed 59,114 OHCA patients in Japan for whom EMS personnel were prohibited from performing TOR at the scene. We evaluated the performance of the conventional BLS rule and investigated the effect of adding post-arrival parameters on predicting outcomes in these patients. The main findings were as follows: First, the conventional BLS-TOR rule demonstrated a specificity of 89% and a PPV of 99% for 30-day mortality. Second, incorporating serum potassium levels, recognised as the strongest independent predictor among various laboratory variables, markedly improved the effectiveness of the conventional BLS-TOR rule. Specifically, adding a potassium threshold increased specificity. In analyses stratified by BLS-TOR status, higher serum potassium levels were consistently associated with reduced odds of survival and a favourable neurological outcome in both groups (Supplementary Tables 2 and 3). Notably, when a more stringent cutoff of ≥8.5 mEq/L was implemented, no survivors with favourable CPC were identified, suggesting that this threshold may represent a clinically meaningful marker of an irreversible state. This is the first study to report that incorporating a simple, rapid, and easily obtainable in-hospital variable can significantly improve the accuracy of the conventional BLS-TOR rule. Additionally, in many healthcare systems, patients who do not meet BLS-TOR criteria are nonetheless transported to hospital, making early in-hospital prognostic assessment particularly important. In this context, objective laboratory parameters that can be obtained immediately after arrival may provide additional information beyond clinical TOR criteria alone.

### 4.2 Comparison with previous studies

Previous international studies have reported how pre-hospital systems and community-level interventions substantially influence outcomes after OHCA.^23^^,^^24^ A recent systematic review reported that bundles of community-based interventions—such as CPR training, public-access Automated External Defibrillator (AED) programs, and dispatcher-assisted CPR—were associated with improvements in bystander intervention rates, survival, and favourable neurological outcomes, although considerable heterogeneity and residual confounding limited the certainty of these findings.^23^ Large observational studies from the United States comparing basic and ALS have further reported that BLS-treated patients may achieve better survival and neurological outcomes than those receiving ALS, potentially owing to differences in scene time, airway management strategies, pharmacologic interventions, and rapid transport to definitive in-hospital care.^24^

In contrast, the structure of the Japanese emergency medical system differs fundamentally. Because pre-hospital TOR is generally not permitted, even patients who meet BLS-TOR criteria are routinely transported to hospital. As a result, this subgroup—considered futile in many Western systems—remains part of the in-hospital resuscitation population and may represent a particularly high-risk cohort. This system-level characteristic is reflected in the marked differences in outcomes observed between patients who did and who did not fulfill the BLS-TOR criteria in our cohort. Overall survival and favourable neurological outcomes in our cohort were 6.0% and 3.1%, respectively. This distinct case mix—driven by routine hospital transport despite meeting TOR criteria—may partly explain the relatively low outcome rates observed in our study and it highlights the need for caution when extrapolating our findings to regions in which pre-hospital termination is routinely practiced.

### 4.3 Clinical interpretation of BLS-TOR subgroups and the role of serum potassium

In this cohort, approximately four-fifths of patients (43,737/59, and 114) met the conventional BLS-TOR criteria. Among them, 18.7% achieved ROSC, 0.9% survived for 30 days, and 0.1% achieved a favourable neurological outcome. Patients who fulfilled the BLS-TOR criteria differed substantially from those who did not, particularly with respect to initial rhythm, witness status, and pre-hospital ROSC. These differences indicate a fundamentally different arrest profile with a markedly lower baseline probability of survival. They also reflect a distinct case mix inherent to healthcare systems in which pre-hospital TOR is not permitted. This contextual difference underscores the need for post-arrival objective markers to support early in-hospital decision-making. These results underscore the limitations of the conventional BLS rule in entirely excluding the possibility of survival and favourable outcomes. The incorporation of serum potassium levels, an easily obtainable parameter in emergency settings, significantly enhanced the discriminatory power of the rule. The 30-day survival rates progressively declined with higher potassium levels, dropping from 0.93% with the conventional TOR criteria alone, to 0.72% at ≥5.0 mEq/L, 0.40% at ≥6.0 mEq/L, and 0.21% at ≥7.0 mEq/L. Hence, serum potassium level is a valuable adjunctive marker that may enhance the prognostic accuracy of the conventional BLS-TOR criteria.

In this context, several TOR rules have been developed, including Goto’s, SOS–KANTO, and KoCARC III rules.^10^^,^[15], [16], [17] Although these rules have demonstrated a high predictive value for 30-day mortality and poor neurological outcomes, they rely primarily on pre-hospital information, the accuracy and completeness of which may vary considerably upon hospital arrival. For instance, the Goto’s rule, the KoCARC III rule, and the BLS-TOR rule all incorporate pre-hospital ROSC as a key determinant. Moreover, both the SOS–KANTO rule and Goto’s rule consider whether a bystander witnessed the arrest. In real-world clinical settings, the collection of accurate pre-hospital data is often incomplete or delayed. In addition, decision-making by EMS personnel, particularly in assessing ROSC, may vary due to time constraints, environmental changes, and variability in adherence to established protocols.^17^ In particular, the inherent uncertainty of pre-hospital information and the complexity of some protocols further complicate the external validation of these rules.^8^ Given these limitations, the incorporation of objective and rapidly obtainable biomarkers upon hospital arrival, such as serum potassium level, may serve as a valuable adjunct for improving prognostic accuracy. This variable is readily available, standardised, and free from subjective interpretation, thereby enhancing the robustness and accuracy of TOR decisions, particularly in cases with borderline or uncertain outcomes, including those with incomplete or unreliable pre-hospital information.

Elevated potassium levels have been reported to be associated with poor neurologic outcomes at one-month post-resuscitation^19^^,^^20^^,^^25^ and prolonged CA and metabolic acidosis.^26^ Hence, severe hyperkalaemia may serve as a marker of extensive ischaemic injury and poor systemic recovery potential. In our study, although the conventional BLS-TOR criteria failed to identify approximately 1% of potential survivors, adding serum potassium markedly improved specificity and reduced the number of missed potential survivors by approximately one-fifth. Therefore, in patients who meet all criteria of the BLS-TOR rule, it may be beneficial to use potassium as a marker to establish the criteria for end-of-life resuscitation. However, serum potassium levels may vary depending on the underlying pathophysiology and clinical course. They can also be affected by factors such as haemolysis. Therefore, to guide critical decision-making, particularly in TOR, potassium levels should be carefully interpreted to avoid false-positive cases due to spurious elevations. Multiple serum potassium measurements should be considered to confirm the accuracy of the initial results.

### 4.4 Implications for clinicians and policy makers

In rapidly aging societies, particularly in developed countries such as Japan, there has been a notable shift in the demographic profile of EMS users, with an increasing proportion of resuscitation candidates being elderly individuals with multiple comorbidities and/or frailty.^27^^,^^28^ Consequently, EMSs increasingly face the challenge of balancing aggressive interventions against poor prognoses and the limited clinical benefits that such interventions provide. The challenge is further compounded by the need for effective resource allocation and ethical considerations, particularly in aging populations and resource-constrained settings. Therefore, in countries where pre-hospital TOR is not permitted, such as Japan, there is an urgent need for objective and rapidly obtainable markers to facilitate early decision-making immediately after hospital arrival. Serum potassium level, which can be measured within minutes, is a practical and reliable indicator of potentially futile resuscitation efforts. Our findings suggest that incorporating serum potassium levels can significantly improve the TOR criteria, better aligning them with healthcare demands and the societal characteristics of aging populations. Future large-scale studies are warranted to validate these findings externally in countries with similar constraints on implementing TOR.

## 5 Limitations

This study has some limitations. First, owing to its retrospective observational design, this study remains vulnerable to residual confounding despite multivariable adjustment, and causal relationships cannot be established. Consequently, the applicability of the findings may be restricted owing to international differences in medical practices and healthcare systems. In addition, the extremely low event rates in this cohort may render conventional diagnostic performance metrics, including sensitivity, specificity, PPV, and AUROC, unstable and should therefore be interpreted with caution. Additionally, this study was based on registry data available up to 2021, which represented the most recent dataset accessible at the time of study approval. As resuscitation practices and post–cardiac arrest care may have evolved since then, the applicability of these findings to current clinical settings may be limited. Second, it remains challenging to determine whether the observed hyperkalaemia is a consequence of CA or the primary cause of the event. Marked hyperkalemia may represent cardiogenic shock, acute kidney injury, severe metabolic acidosis, or extensive tissue damage. Although blood samples were collected as early as possible after hospital arrival, serum potassium levels may have been influenced by pre-hospital and in-hospital interventions, including fluid administration and pharmacological treatments. Third, local medical control protocols and post-hospitalisation care may have differed between facilities, potentially leading to the selection of patients with more favourable neurological outcomes. Therefore, some cases in which resuscitation was terminated early due to a do-not-attempt-resuscitation order after hospital arrival or during transport, as well as cases in which invasive interventions were withheld, resulting in the patient’s death, could not be excluded. Fourth, although serum potassium emerged as the strongest additional predictor in our analyses, this finding should be regarded as exploratory and hypothesis-generating rather than sufficient to establish a definitive new TOR rule. Important clinical variables that are not captured in the registry may further refine prognostication and should be incorporated in future studies.

### 5.1 Areas for future research

Prospective validation in independent cohorts will be required before considering any modification of existing TOR criteria. Despite these limitations, our findings suggest that serum potassium levels may serve as a valuable marker for post-arrival TOR decisions.

## 6 Conclusion

Post-arrival in-hospital variables may enhance the accuracy of rule-based TOR decisions in settings where pre-hospital TOR is not permitted. Although serum potassium appears to be a potentially useful adjunctive prognostic marker, a potassium level >8.5 mmol/L on arrival was associated with no favourable neurological outcomes in our cohort. Further prospective research is required to validate these findings and to identify additional practical and readily available parameters before considering any modification of existing TOR criteria.

## CRediT authorship contribution statement

Akira Suekane and Wataru Takayama contributed to conceptualisation, data curation, formal analysis, writing- original draft. Koji Morishita contributed to writing- review and editing. All the authors have read the manuscript, contributed to the interpretation of the results, and approved the final version for submission.

## Data sharing statement

The datasets analysed in this study are not publicly available due to privacy issues but are available from the corresponding author upon reasonable request.

## Funding

This research did not receive any specific grant from funding agencies in the public, commercial, or not-for-profit sectors.

## Conflicts of interest

The authors declare that they have no known competing financial interests or personal relationships that could have appeared to influence the work reported in this paper.
