# Supplementary material for: Reevaluating basic life support termination criteria using post-arrival data in Japanese out-of-hospital cardiac arrest patients
Source: Crit Care Resusc. 2026 May 12;28(2):100186. doi: 10.1016/j.ccrj.2026.100186 (PMC13195281; doi:10.1016/j.ccrj.2026.100186)
Supplement: Multimedia component 1 [file mmc1.docx]

| **Supplementary Information**  **Supplementary Table 1. Univariate analyses of post-arrival factors associated with 30-day survival** | | | | |
| --- | --- | --- | --- | --- |
| **Variables** | **Overall** | **Not fulfilled**  **the BLS-TOR rule** | **Fulfilled the BLS**  **-TOR rule** | **p** |
|  | n = 59,114 | n = 15,377 | n = 43,737 |  |
| **pH** | 7.0 [7.0, 7.0] | 7.0 [7.0, 7.0] | 7.0 [7.0, 7.0] | **<0.01** |
| **PaCO_2_** | 88.0 [67.0, 114.0] | 78.0 [58.0, 98.0] | 91.0 [69.0, 119.0] | **<0.01** |
| **PaO_2_** | 31.0 [17.0, 63.0] | 43.0 [20.0, 81.0] | 29.0 [16.0, 58.0] | **<0.01** |
| **HCO_3_** | 15.0 [12.0, 19.0] | 16.0 [12.0, 20.0] | 15.0 [11.0, 19.0] | **<0.01** |
| **BE** | -19.0 [-24.0, -13.0] | -17.0 [-22.0, -12.0] | -19.0 [-24.0, -14.0] | **<0.01** |
| **Lactate** | 125.0 [92.0, 162.0] | 107.0 [78.0, 138.0] | 131.0 [96.0, 171.0] | **<0.01** |
| **Glu** | 209.0 [114.0, 309.0] | 258.0 [169.0, 344.0] | 194.0 [104.0, 296.0] | **<0.01** |
| **Sodium** | 140.0 [137.0, 144.0] | 140.0 [137.0, 143.0] | 140.0 [136.0, 144.0] | 0.23 |
| **Potassium** | 6.0 [5.0, 8.0] | 5.0 [4.0, 6.0] | 7.0 [6.0, 9.0] | **<0.01** |
| Abbreviations: BLS, Basic life support; TOR, Termination of resuscitation; BE, Base excess; CI, Confidence interval; Glu, Glucose | | | | |

| **Supplementary Table 2. Multivariate logistic regression analyses of post-arrival factors in patients who fulfill the BLS-TOR rule** | | | | | | | | |
| --- | --- | --- | --- | --- | --- | --- | --- | --- |
|  | **Survival at 30 days** | | | | **Favorable CPC at 30 days** | | | |
| **Variables** | **Odds ratio** | **Lower 95% CI** | **Upper 95% CI** | **p** | **Odds ratio** | **Lower 95% CI** | **Upper 95% CI** | **p** |
| **pH** | 1.49 | 0.24 | 10.07 | 0.70 | 2.31 | 0.04 | 114.31 | 0.78 |
| **PaCO_2_** | 1.01 | 1.00 | 1.02 | 0.01 | 1.01 | 0.98 | 1.03 | 0.55 |
| **PaO_2_** | 1.01 | 1.00 | 1.01 | **<0.01** | 1.01 | 1.00 | 1.01 | 0.04 |
| **HCO_3_** | 0.99 | 0.95 | 1.04 | 0.70 | 1.01 | 0.86 | 1.13 | 0.92 |
| **BE** | 1.02 | 0.98 | 1.05 | 0.24 | 0.95 | 0.84 | 1.06 | 0.45 |
| **Lactate** | 0.99 | 0.99 | 1.00 | 0.01 | 0.98 | 0.97 | 1.00 | **<0.01** |
| **Glu** | 1.00 | 1.00 | 1.00 | 0.61 | 1.00 | 0.99 | 1.00 | 0.39 |
| **Sodium** | 0.98 | 0.96 | 1.01 | 0.11 | 0.95 | 0.90 | 1.01 | 0.08 |
| **Potassium** | 0.48 | 0.42 | 0.54 | **<0.01** | 0.54 | 0.37 | 0.76 | **<0.01** |
| Abbreviations: BLS, Basic life support; TOR, Termination of Resuscitation; BE, Base Excess; CI, Confidence interval; Glu, Glucose; CPC, Cerebral Performance | | | | | | | | |

| **Supplementary Table 3. Multivariate logistic regression analyses of post-arrival factors in patients who did not fulfill the BLS-TOR rule** | | | | | | | | |
| --- | --- | --- | --- | --- | --- | --- | --- | --- |
|  | **Survival at 30 days** | | | | **Favorable CPC at 30 days** | | | |
| **Variables** | **Odds ratio** | **Lower 95% CI** | **Upper 95% CI** | **p** | **Odds ratio** | **Lower 95% CI** | **Upper 95% CI** | **p** |
| **pH** | 1.22 | 0.33 | 4.12 | 0.78 | 1.23 | 0.21 | 5.60 | 0.81 |
| **PaCO_2_** | 0.99 | 0.98 | 0.99 | 0.55 | 0.96 | 0.95 | 0.97 | **<0.01** |
| **PaO_2_** | 1.00 | 1.00 | 1.00 | 0.04 | 1.00 | 1.00 | 1.00 | 0.41 |
| **HCO_3_** | 1.00 | 0.97 | 1.03 | 0.92 | 1.00 | 0.96 | 1.04 | 0.98 |
| **BE** | 1.02 | 1.00 | 1.04 | 0.45 | 1.01 | 0.98 | 1.04 | 0.68 |
| **Lactate** | 1.00 | 0.99 | 1.00 | **<0.01** | 0.99 | 0.99 | 1.00 | **<0.01** |
| **Glu** | 1.00 | 1.00 | 1.00 | 0.39 | 1.00 | 1.00 | 1.00 | 0.05 |
| **Sodium** | 0.99 | 0.97 | 1.01 | 0.08 | 1.01 | 0.99 | 1.04 | 0.29 |
| **Potassium** | 0.56 | 0.51 | 0.61 | **<0.01** | 0.51 | 0.45 | 0.58 | **<0.01** |
| Abbreviations: BLS, Basic life support; TOR, Termination of Resuscitation; BE, Base Excess; CI, Confidence interval; Glu, Glucose; CPC, Cerebral Performance | | | | | | | | |

| **Supplementary Table 4. Predictive performance of potassium for unfavorable CPC at 30 days in patients who fulfilled the BLS**  **BLS-TOR criteria** | | | | |
| --- | --- | --- | --- | --- |
| **TOR rules** | **The BLS TOR rule** | **The BLS TOR rule and potassium** | | |
| **Criteria** | **Not witnessed by EMT**  **No prehospital shock**  **No prehospital ROSC** | **Not witnessed by EMT**  **No prehospital shock**  **No prehospital ROSC**  **Potassium** | | |
| **Threshold of potassium, mEq/L** |  | > 5.0 | > 6.0 | > 7.0 |
| **N** | 43,737 | 20,219 | 16,819 | 12,800 |
| **Favorable CPC at 30 days (%)** | 53 (0.12) | 15 (0.07) | 7 (0.04) | 4 (0.03) |
| **Sensitivity (95%CI)** | 0.76 (0.76-0.77) | 0.66 (0.66-0.67) | 0.55 (0.55-0.56) | 0.42 (0.42-0.43) |
| **Specificity (95%CI)** | 0.97 (0.96-0.98) | 0.99 (0.98-0.99) | 1.00 (0.99-1.00) | 1.00 (0.99-1.00) |
| **PPV (95%CI)** | 1.00 (1.00-1.00) | 1.00 (1.00-1.00) | 1.00 (1.00-1.00) | 1.00 (1.00-1.00) |
| **NPV (95%CI)** | 0.12 (0.11-0.12) | 0.11 (0.11-0.12) | 0.11 (0.11-0.12) | 0.07 (0.07-0.07) |
| **Area under the ROC curve (95%CI)** | 0.87 (0.86-0.87) | 0.83 (0.82-0.83) | 0.77 (0.77-0.78) | 0.71 (0.71-0.71) |
| Abbreviations: BLS, Basic life support; TOR, Termination of resuscitation; ROSC: Return of spontaneous circulation; CI, Confidence interval; CPC, Cerebral performance category; PPV, Positive predictive value; NPV, Negative predictive value; ROC, Receiver operating characteristic curve; EMT, Emergency medical team | | | | |

**
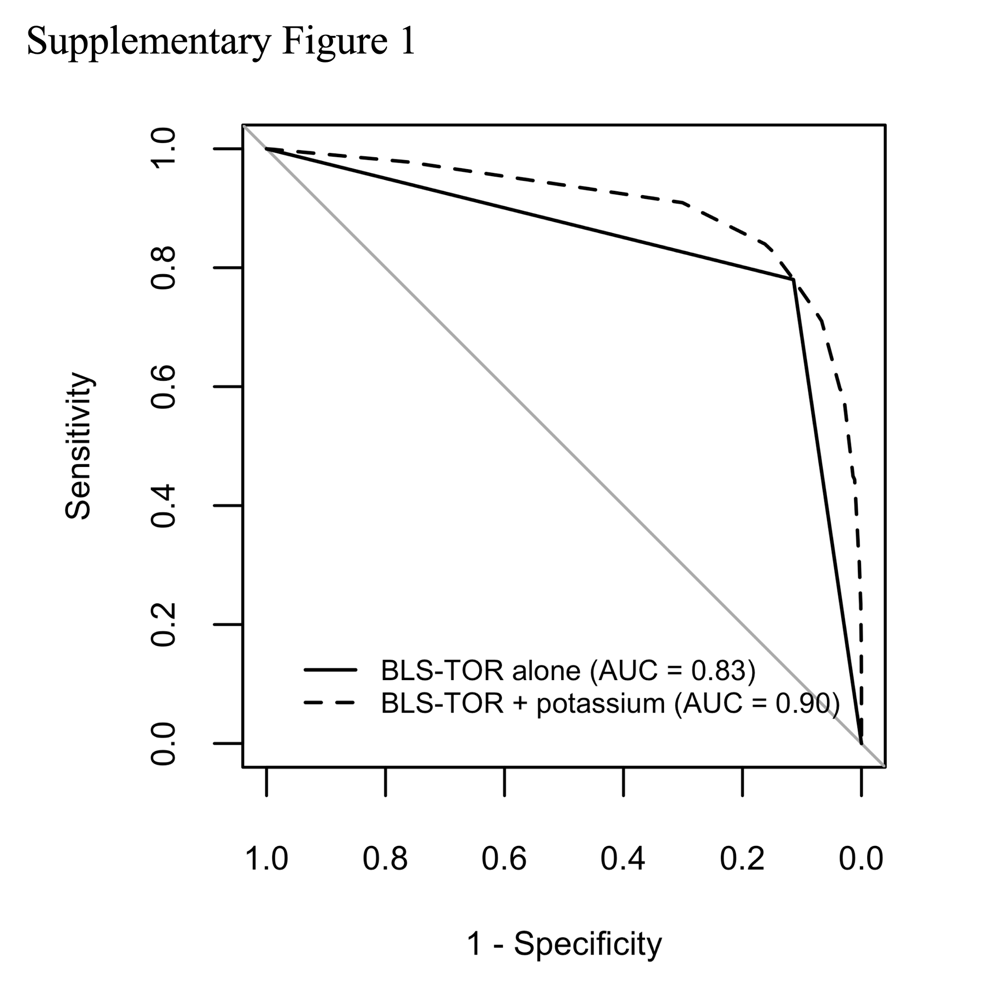
**

**Supplementary Figure 1.** Receiver operating characteristic curves comparing prediction of 30-day mortality using the basic life support termination-of-resuscitation (BLS-TOR) rule alone and the BLS-TOR rule plus admission serum potassium level. The solid line represents the BLS-TOR rule alone, and the dashed line represents the model including potassium. Areas under the curve (AUCs) are shown in the figure.
